# Unilateral Facial Talon Cusp on Maxillary and Mandibular Second Molar: A Case Series

**DOI:** 10.7759/cureus.111760

**Published:** 2026-06-29

**Authors:** Ramachandra Reddy Gowda Venkatesha, Karthik Rajaram Mohan, Mathi Vadhana Ramasamy Shanmugam Vijayan, Saramma Mathew Fenn

**Affiliations:** 1 Oral Medicine and Radiology, Vinayaka Mission's Sankarachariyar Dental College, Vinayaka Mission's Research Foundation (Deemed to be University), Salem, IND

**Keywords:** facial talon cusp, mandibular molar, occlusal interferences, pit and fissure sealants, dental caries

## Abstract

*Mandibular Talon cusp* is a rare developmental tooth anomaly characterized by an extra cusp-like projection on the buccal or lingual surface of mandibular molar teeth. An oral physician often overlooks the Talon cusp during clinical examination, as it is often asymptomatic clinically. Rarely does it cause occlusal interference or plaque build-up on the tooth, resulting in periodontitis or dental caries. Such Talon cusp is important in diagnosis as it can cause occlusal interference and plaque build-up, making teeth more prone to caries. We report a rare case series of such a talon cusp on the buccal aspect of the left mandibular second molar in a 21-year-old male and the right maxillary second molar in a 41-year-old male.

## Introduction

The mandibular Talon cusp contains varying amounts of enamel, dentin, and pulp tissue [1]. It is known by other names such as cusped cingulum, dens evaginatus, evaginated odontome, supernumerary cusp, hyperplastic cingulum, and palatal cusp [1]. It was named Talon Cusp since it resembles an eagle's talon. Permanent teeth are affected more than primary teeth and among the male gender. Talon cusp arises from the morpho-differentiation stage of tooth development. The reported incidence was 0.001%, and the prevalence of Talon cusp was 2.95 % and 0.58% among the north and south Indian populations [2]. The prevalence of the Talon cusp in the primary dentition was 0.6% in Japan [3]. The prevalence of Talon cusp ranges from maxillary lateral incisors (54.8%), maxillary central and canine (16.12%), and mandibular central incisors (6.45%) [4].

## Case presentation

Case 1

**Figure 1:**
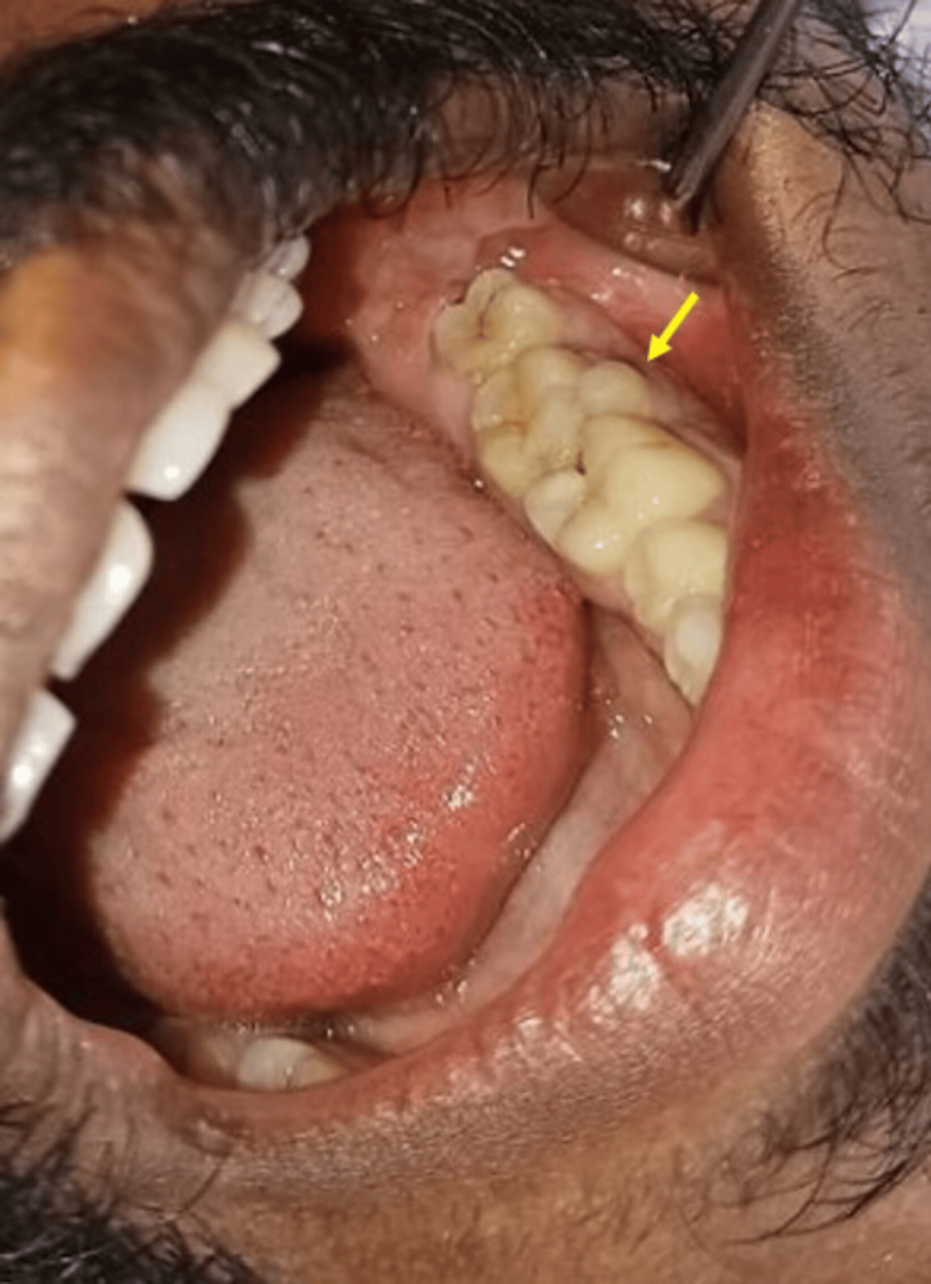
Talon cusp on mandibular second molar

A 21-year-old male reported to our dental department for a routine dental checkup. Intraoral clinical findings revealed a talon-cusp-like structure resembling the shape of an eagle's talon on the buccal aspect near the occlusal surface in the mandibular second molar #37. The radiograph was not provided since the Talon cusp was asymptomatic (Figure 1).

Case 2

**Figure 2:**
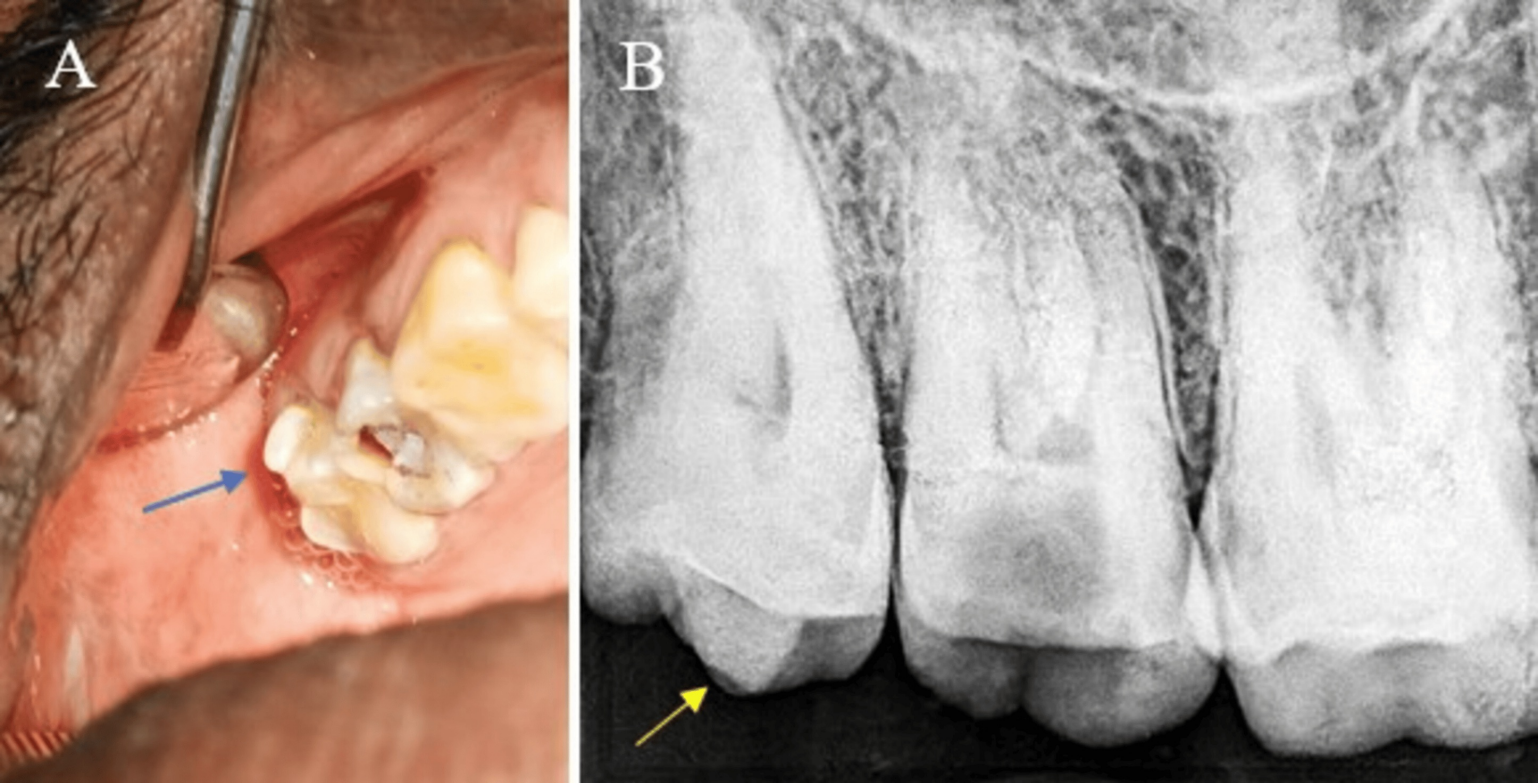
A. Intraoral examination revealed a Talon-cusp on the buccal surface of the Maxillary second molar. B. Intraoral periapical radiograph revealed a Talon-cusp-like structure in the crown of the Maxillary second molar

A 41-year-old male reported a chief complaint of food lodgement in the decayed right maxillary molar region. Intraoral clinical examination revealed a cusp-like hard mass on the buccal surface of the crown of the right maxillary second molar (Figure 2A). Intraoral periapical radiograph revealed an extra cusp-like structure on the crown of the right maxillary second molar (Figure 2B).

Clinical significance

Talon cusp is often overlooked and goes unnoticed by a dentist during the clinical examination. Talon cusp can cause occlusal interference, resulting in plaque build-up around the crown and dental caries or periodontal diseases. Talon cusp causes abnormal occlusal stresses to the underlying periodontium, resulting in trauma from occlusion. It also causes the wedging of food particles into the periodontium, resulting in the early occurrence of periodontal diseases [3-5].

## Discussion

Talon cusp is a supernumerary extra-cusp-like structure extending from a cementoenamel junction of varying depths [2,3]. Talon cusp is believed to arise from dental lamina hyperactivity or due to enamel lamina folding during the morpho-differentiation stage of tooth development [4]. The occurrence of Talon-cusp in mandibular posterior teeth is very rare [5]. A new scoring system for Talon cusp (Table 1) [3]. The new scoring system for Talon cusp is enumerated (Table 2).

**Table 1:** Classification of Talon Cusp Table created with Permission from Author [2] F: Facial, B: Buccal, L: Lingual, P: Palatal

Type of talon cusp	Name of talon cusp	Clinical features
Type I	Talon	A morphologically well-delineated additional cusp that prominently projects from the palatal surface usually or facial surface rarely in primary or permanent maxillary central lateral or canine tooth and extends at least half the distance from the incisal edge to the cementoenamel junction.
Type II	Semi talon	An additional cusp of a millimeter or more extending less than half the distance from the incisal edge to the cementoenamel junction. It may stand away from the rest of the crown or blend with the palatal surface.
Type III	Trace talon	Prominent or enlarged cingula and its variations, that is, bifid, tubercle-like, or conical.
Based on the surface involved	F	
	L/P	
	FL/P	
Comprehensive integrated classification system of talon cusp	Teeth involved in Fédération Dentaire Internationale system	
	U or BL	
	Degree of cusp formation ranging from Type I to Type III	
	Surface involved ranging from F to FL/P	

**Table 2:** New scoring system for Talon cusp Table created with Permission from author [3]

Locus / Stages	Location of Talon cusp
Locus 1	corresponds to palatal and lingual talon cusp
Stage 1	trace talon, enlarged cingula and their variation
Stage 2	linked semi-talon: 1 mm or more additional totally linked cusps extending less than half the length from the cemento-enamel junction to the incisal edge
Stage 3	free semi-talon: 1 mm or more additional free cusp extending less than half the length from the cemento-enamel junction to the incisal edge
Stage 4	linked true talon: additional totally linked cusp extending more than half the length from the cemento-enamel junction to the incisal edge
Stage 5	free true talon: additional free cusp extending more than half the length from the cemento-enamel junction to the incisal edge
Locus 2	Corresponds to Labial talon cusp
Stage 1	slight talon: vertical ridge not reaching the incisal edge or the cemento-enamel junction
Stage 2	moderate smooth talon: vertical ridge with smooth connection to the labial surface reaching the incisal edge but not the cemento-enamel junction
Stage 3	moderate grooved talon: vertical ridge with deep grooved connection to the labial surface reaching the incisal edge but not the cemento-enamel junction
Stage 4	extreme smooth talon: vertical ridge with smooth connection to the labial surface reaching the incisal edge and the cemento-enamel junction
Stage 5	extreme grooved talon: vertical ridge with deep grooved connection to the labial surface reaching the incisal edge and the cemento-enamel junction
Locus 3	corresponds to composite features, both palatal and labial, and their corresponding stages
Locus 1	Stage 1, 2, 3, 4 or 5
Locus 2	Stage 1, 2, 3, 4 or 5
Locus 3	Stage 1, 2, 3, 4 or 5 for palatal, lingual talon/stage 1, 2, 3, 4 or 5 for labial talon.

Incidence and prevalence

The incidence of Talon cusp was 0.04- 8% [4]. The prevalence of Talon cusp among various populations and Malaysian (5.2%), Hungarian (2.5%), Jordanian (2.4%), France (1.67%), and Mexican (0.6%) [5,6]. Talon cusp has no gender predilection [6]. The occurrence of Talon cusp in mandibular posterior teeth is very rare [7]. Talon cusp is reported in patients with Ellis-van Creveld, Rubinstein-Taybi syndrome and Mohr syndrome (orofacial - digital II), Encephalotrigeminal angiomatosis or Sturge-Weber syndrome, and other tooth conditions such as impacted teeth, taurodontism, and compound odontoma [7].

Etiology

Talon cusps on permanent teeth have been reported in patients with Mohr syndrome, Sturge-Weber syndrome, and RubinsteinTaybi syndrome, while talon cusps in primary teeth have been reported in patients with cleft lip and palate, hypomelanosis of Ito and Ellis-van Crevald syndrome [4]. A 4-year-old girl affected by hypomelanosis of Ito reportedly had multiple talon cusps affecting three primary maxillary incisors and, a primary mandibular right lateral incisor and also the maxillary permanent successors [4]. However, it was mentioned that, in contrast to ordinary talon cusps, the outgrowths were of hamartomatous origin [4]. It has been suggested that talon cusp might occur due to an outward folding of the inner enamel epithelial cells and a transient focal hyperplasia of the mesenchymal dental papilla. Talon cusp has also been viewed as one end of a range of hyperactivity of the dental lamina, with the other end being a supernumerary tooth.

Moreover, it has been suggested that a talon cusp may result from fusion of a normal and a supernumerary tooth. The fact that the talon cusp in the permanent dentition usually affects the maxillary lateral incisors while in the primary dentition, the maxillary central incisors are affected suggests that the etiological factors for permanent and primary teeth may differ. The various reports of talon cusps on primary maxillary central incisors affecting two pairs of twins from different families and talon cups in permanent incisors of cousins, siblings, or parents may imply that talon cusp has a genetic etiology. It is most likely that the talon cusp is determined by a multifactorial etiology involving both genetic and environmental factors [4].

Talon cusp does not need dental treatment unless it causes occlusal interference or plaque build-up, resulting in dental caries [6]. The application of pit-and-fissure sealants prevents dental caries in the mandibular molar. If the mandibular talon cusp interferes with occlusion, coronoplasty is advised, followed by topical fluoride application to prevent dentinal hypersensitivity. Correction of the malaligned adjacent opposing tooth is advised if the talon cusp causes malocclusion of opposing teeth [3-6]. Srinivasan B et al. reported an occurrence of the Talon cusp in the maxillary lateral incisor that hindered the eruption of the maxillary canine [7]. Oredugba FA stated the presence of a talon cusp is not always an indication for dental treatment unless it is associated with problems such as compromised aesthetics, occlusal interference, tooth displacement, caries, periodontal problems, or irritation of the soft tissues during speech or mastication. The management and treatment outcome of talon cusp depends on the size, presenting complications, and patient cooperation [8]. Karjodkar FR and Gupta A reported a case of Talon cusp in the mandibular lateral incisor that caused a periodontal problem. The talon cusp has not been reported as an integral part of any specific syndrome, although it appears to be more prevalent in patients with Rubinstein-Taybi syndrome, Mohr syndrome, and Sturge-Weber syndrome [9]. Chaitra TR et al. reported cases of Talon cusp in mandibular central incisors stated that early diagnosis and management of talon's cusp is important for the sake of preventing occlusal interference, compromised aesthetics, carious developmental grooves, periodontal problems due to excessive occlusal forces and irritation of the tongue during speech and mastication and a talon cusp can be left untreated if it lies dormant without causing any harm [10]. Segura-Egea JJ et al. reported a Talon cusp in the maxillary lateral incisor that caused occlusal interference due to occlusal trauma from uneven occlusal forces generated by Talon cusp and resulting in reversible apical periodontitis in the opposing teeth [11]. No Talon cusp was reported in the left mandibular second molar and right maxillary second molar.

## Conclusions

Talon cusp is often asymptomatic and usually goes unnoticed during routine clinical examination. Talon cusp does not need any treatment unless it causes problems like occlusal interference, dental caries, or plaque build-up resulting in periodontitis and can be treated by coronoplasty in case of occlusal interference, application of pit and fissure sealants and topical application of fluoride varnish if dental caries formation occurs in talon cusp.
